# Family income is associated with quality of life in patients with chronic kidney disease in the pre-dialysis phase: a cross sectional study

**DOI:** 10.1186/s12955-015-0390-6

**Published:** 2015-12-21

**Authors:** Camila Foresti Lemos, Marcelo Palmeira Rodrigues, Joel Russomano Paulo Veiga

**Affiliations:** University of Brasilia (UNB), Post-Graduate College of Medicine (UNB), Master (Msc) and Nurse Specialist in Nephrology, Campus Darcy Ribeiro, North Wing, Brasilia, 70910900 DF Brazil; University of Brasilia (UNB), Laboratory of pneumology, Doctor (MD, PhD) and Adjunct professor, College of Medicine (UNB), Campus Darcy Ribeiro, North Wing, Brasília, DF Brazil; University of Brasilia, Laboratory of Nephrology, Doctor (MD, PhD) and Associate professor, College of Medicine (UNB), Campus Darcy Ribeiro, North Wing, Brasília, DF Brazil

**Keywords:** Quality of life, Family income, Chronic kidney disease, Pre-dialysis

## Abstract

**Background:**

Chronic kidney disease (CKD) is a condition of high prevalence in the general population mainly due to hypertension and diabetes mellitus. It is often associated with a high prevalence of complications and worse quality of life. The main objective of this study is to evaluate quality of life (QOL) using the generic instrument SF-36 in patients with CKD in pre-dialysis and identify the possible influence of the degree of renal function, hemoglobin level, age, gender, family income and level of education on QOL.

**Methods:**

A cross-sectional study was conducted and included 170 individuals (83 men) with a mean age of 57 ± 15 years who met the inclusion criteria and answered the SF-36. Laboratory tests and clinical and demographic data were obtained, and the glomerular filtration rate was estimated using the CKD-EPI formula.

**Results:**

The degree of renal function did not influence QOL. Women had lower scores in functional capacity, physical aspects, pain, and mental health. Patients younger than 47 years old showed better QOL in the functional capacity; however, their QOL was worse in terms of social aspects. Subjects with an income higher than 5.1 times the minimum wage had better QOL in the functional capacity, pain, social, physical and emotional roles, and mental health. Hemoglobin levels and education did not globally influence QOL.

**Conclusion:**

Gender and age influenced QOL, but family income was the most important factor affecting QOL (6 out of 8 domains investigated by SF-36) in this sample of 170 individuals with CKD in pre-dialysis. These findings suggest that many efforts should be made to reduce the effect of these factors on quality of life in patients with CKD and reinforce the need for longitudinal studies and intervention.

## Background

The prevalence of chronic kidney disease (CKD), defined by reduced glomerular filtration rate (GFR) and/or presence of proteinuria [1], is increasing worldwide due to an aging population and a global epidemic of diabetes [2]. In its final evolutionary stage, there is the need for permanent dialysis or a kidney transplant, which leads to very high health care costs for many countries.

Also, as described in previous work, CKD increases cardiovascular risk [3, 4] and leads to loss of quality of life in terms of health [5–9].

Hence, some studies have shown that quality of life (QOL), assessed by internationally validated questionnaires, is worse in individuals with CKD compared to those without renal disease [10], and that the morbidity and mortality in end-stage disease are influenced by timing and quality care before the start of dialysis [8, 11]. A systematic review of the QOL scores of these patients as a parameter of treatment adequacy is recommended [1] and it is also suggested that interventions in pre-dialysis are useful and can influence the clinical evolution in the later stages of the disease [1, 8].

However, information on the QOL of patients undergoing conservative CKD treatment and the socio-demographic factors that may influence QOL is limited.

The present study aims to assess the QOL of pre-dialysis CKD patients by means of the SF-36, and to investigate the possible influence of CKD staging, hemoglobin, age, family income, gender, and educational level on the QOL of these individuals.

## Methods

This is a cross-sectional study of outpatients with pre-dialysis CKD monitored at Hospital Universitário de Brasilia (HUB) and Hospital de Base do Distrito Federal (HBDF) conducted from February 2011 to November 2012 (20 months).

Inclusion criteria were: 1) Having been diagnosed with CKD, 2) Not having undergone a kidney transplant, 3) Aged over 18 years; 5) agreeing to participate in the study by signing a consent form.

Exclusion criteria were: 1) Neurological disorders that may affect the implementation of tests; for instance, a cerebrovascular accident (CVA); 2) Degree of literacy and inability to understand the research objectives, 3) Patients with comorbidities such as active cancer, infectious or inflammatory diseases; 4) Mini-Mental State Examination (MMSE) score lower than 14.

On the day of the interview, the participants’ socio-demographic data were obtained, including name, address, phone number, age, gender, level of education, family income, race, comorbidities (diseases that accompany CKD), etiology of CKD, current occupation, leisure activities, weight, height, body mass index (BMI), continuous use medication, list of symptoms, blood pressure (BP), and laboratory tests. We also recorded the knowledge of CKD patients about the disease and outpatient follow-up time.

Their cognitive function was assessed by means of the Mini Mental test, which is reliable and valid and is a broad measure of the global cognitive status based on the Mini-Mental State Examination (MMSE) [12]. It includes assessment guidance (range = 0–10), registration and digit span (range = 0–13), attention and calculation (range = 0–7), general knowledge (range = 0–5), recovery (range = 0–3), language (range = 0–17), and construction (range = 0–2) [12].

Later, to assess QOL, the SF-36 questionnaire (*Medical Outcomes Study 36 – Item Short – Form Health Survey*), translated and validated for the Portuguese language, [17] was used. This questionnaire was chosen for its reliability, frequency of use and ease of application. It consists of 36 items divided into eight dimensions: physical functioning (ten items), physical function (four items), emotional function (three items), social function (two items), emotional well-being (five items), pain (two items), energy/fatigue (four items), and general health (five items) [13]. Some domains assess the physical aspect while others evaluate the mental aspect, and this yields the physical (PS) and mental health (MS) summary scales.

Data were collected from patients of both genders, considering the inclusion and exclusion criteria. We invited 185 individuals who met the inclusion criteria. Out of these, five interrupted the interview and did not complete the SF-36, four did not agree to sign the consent form, three had been diagnosed with comorbidities listed in the exclusion criteria and three obtained a mini-mental score lower than 14. One hundred and seventy patients completed the study. Disease staging was done through the estimated GFR calculated from serum creatinine using the CKD-EPI equation [14] and CKD was classified according to the National Kidney Foundation [1].

Data collection began only after approval of the research by the Ethics Research Committee of the College of Medicine, University of Brasilia, and it was carried out by a single interviewer. This research offered no risks to participants and all patients gave written informed consent to participate.

Blood samples collected at 12-hour fasting determined concentrations of creatinine, urea, hemoglobin, total cholesterol (TC), high density lipoprotein (HDL), low density lipoprotein (LDL), triglycerides, C reactive protein (CRP), calcium, phosphorus, parathyroid hormone (PTH), glucose, sodium, potassium, and uric acid.

The 10-year cardiovascular risk (Framingham risk score) was calculated [4].

### Statistical analysis

A priori, a minimum number of 160 patients was established, assuming the effect size “f” of 0.25 for the outcome, which represents a moderate effect, considering five analysis groups, plus 70 % power and a value of α equal to 5 %.

The normality of each continuous variable was evaluated by visual observation of each quantile-quantile graph (Q-Q plot); normal distribution occurs if the points formed by the sample quantiles are aligned on a straight slope.

Continuous variables are presented as mean ± standard deviation (SD) and categorical variables as percentages. The “t” test and the Pearson correlation test were used to search for possible correlations between the different parameters observed and the domains of SF-36. Gender, age, level of education, income, and hemoglobin were associated with different QOL domains, being candidates for a multivariate analysis (MANCOVA). CKD staging was included in the analysis due to its influence on QOL in previous studies.

Considering the assessment of QOL as a combination of all the different domains of SF-36, a MANCOVA was performed. It had as dependent variables the 8 domains of SF-36 and as independent variables CKD staging, age, gender, income, level of education, renal function, and hemoglobin, one at a time. The analysis was also controlled for gender and age.

The MANCOVA was used along with Wilks’ lambda test to evaluate the relationship among variables. Two conditions for the analysis were observed: if the data for each dependent variable (SF-36) were normally distributed; if Box’s “M” test indicated there was no violation of the homogeneity assumption of the variance-covariance matrices. The SF-36 domains were considered dependent variables and age, gender, CKD staging, level of education, income, and hemoglobin, one at a time, were considered explanatory variables. Gender plus age were considered covariates.

Continuous explanatory variables were turned into categorical variables as a requirement for the MANCOVA. Thus, hemoglobin was divided into three categories: less than or equal to 9 g/dL; greater than or equal to 13 g/dL; between 13 and 9 g/dl g/dL. Similarly, the limits of 47 and 60 years (25th and 50th percentiles, respectively) were used for age; for income, values from 1 to 5 minimum wages were considered; in terms of level of education, values from 3 to 11 years were used.

The SPSS software version 20 was used to assess the data. *P* < 0.05 was considered statistically significant.

## Results

The study included a sample of 170 CKD patients aged 57.38 ± 15.74 (mean ± SD), out of these, 51 % were female and 55 % white. Most of them had a partner; only 39 % were single, separated and/or widowed. In terms of level of education, there was a variation from 0.5 to 20 years of study; 55 % of subjects had studied at least 5 or more years. Family income averaged 3.34 times the minimum wage; the maximum value was 10 minimum wages and the minimum, 1; interquartile range was 5-1 and standard deviation, 2.33.

When participants were asked about the triggering factor of CKD, 30.58 % of them did not know what it was. In this case, the information was obtained from medical records and it was also used to confirm the patients’ report. Among the associated diagnoses, hypertension (HBP) (88 %) and diabetes mellitus (DM) (31 %) were found, and most diabetics were also hypertensive. The average weight was 69.65 kg (±16.57), height was 1.61 m2 (± 0.09), body surface area of 1.75 m2 (± 0.22), and Body Mass Index (BMI) of 26.35 kg/m2 (± 5.81). Fatigue, cramps and nausea were, respectively, the most frequently reported complaints. Most individuals were sedentary (62.94 %), but they had a leisure activity (61.76 %) and had been in outpatient medical care for more than one year (65.29 %). The 10-year cardiovascular risk, according to the Framingham risk score, was higher for men, and 40.58 % showed intermediate to high risk.

Table 1 shows the overall average of laboratory tests, BMI, Systolic Blood Pressure (SBP), Diastolic Blood Pressure (DBP), age, cardiovascular risk (f*ramingham risk*), family income and staging of the 170 individuals according to gender.

**Table 1:** Anthropometrics, physical data, cardiovascular risk, laboratory tests and family income of 170 individuals according to gender

Parameters	Indivíduals		P#
	Male	Female	
	*N* = 83	*N* = 87	
Age (years)	61.19 ± 14.89	53.74 ± 15.74	0.002
BMI (kg/m^2^)	26.39 ± 5.12	26.44 ± 5.84	0.945
SBP (mmHg)	131.81 ± 24.25	130.17 ± 26.30	0.672
DBP (mmHg)	82.56 ± 15.35	80.00 ± 13.16	0.243
Framingham risk	13.90 ± 8.96	5.73 ± 6.10	<0.001
GFR (ml/min/1.73 m^2^)	33.39 ± 18.75	30.94 ± 20.80	0.422
Creatinine (mg/dL)	2.75 ± 1.82	2.62 ± 1.80	0.643
Hemoglobin (g/dL)	13.39 ± 2.06	12.08 ± 1.82	<0.001
Total cholesterol (mg/dL)	184.01 ± 47.72	203.52 ± 47.74	0.009
HDLc (mg/dL)	40.25 ± 15.53	46.24 ± 14.20	0.012
LDLc (mg/dL)	107.75 ± 41.73	120.54 ± 45.25	0.076
Triglycerides (mg/dL)	172.88 ± 91.43	170.68 ± 118.73	0.897
Calcium (mg/dL)	9.05 ± 1.11	9.13 ± 0.65	0.576
Phosphorus (mg/dL)	4.04 ± 1.10	4.24 ± 0.96	0.240
PTH (pg/mL)	140.35 ± 208.90	236.93 ± 259.81	0.080
Proteinuria (mg/day)	91.63 ± 164.62	97.27 ± 237.38	0.928
Glucose (mg/dL)	99.33 ± 29.96	101.00 ± 27.52	0.714
Sodium (mEq/L)	137.75 ± 7.26	139.54 ± 3.32	0.043
Potassium (mEq/L)	4.65 ± 0.85	4.61 ± 0.65	0.735
Uric acid (mg/dL)	7.53 ± 1.92	6.90 ± 1.54	0.026
Ferritin (ug/L)	164.78 ± 189.67	260.03 ± 368.29	0.138
Income (minimum wage)	3.88 ± 2.21	2.81 ± 2.26	0.002

*BMI* Body Mass Index, *SBP* Systolic Blood Pressure, *DBP* Diastolic Blood Pressure, *eGFR* estimated glomerular filtration rate (CKD-EPI), *TC* total cholesterol, *HDLc* high density lipoprotein cholesterol, *LDLc* low density lipoprotein cholesterol, *PTH* parathyroid hormone

#T test

According to the estimated value of GFR from serum creatinine and the use of the CKD-EPI formula, the interviewed participants were in stages I/II (*n* = 18), III (*n* = 56), IV (*n* = 64) and V (*n* = 32). As their renal function deteriorated, from the initial stages to stage V, it was observed that hemoglobin significantly decreased, and phosphorus, potassium, uric acid and PTH increased.

With regard to gender, women were younger (53.74 ± 15.74 versus 61.19 ± 14.89, *p* = 0.002), had reduced hemoglobin, lower uric acid and higher TC, HDL and sodium and had lower family income (2.81 ± 2.26 versus 3.88 ± 2.21, *p* = 0.002) than men.

Table 2 shows the dimensions of the SF-36 obtained from the 170 patients with CKD according to gender-adjusted estimates by age. It shows that gender influenced QOL. The men with CKD in this study had a better quality of life than women. Men with CKD had better functional capacity, mental health, pain domain and physical aspects.

**Table 2:** Different domains of SF-36 obtained from 170 patients with CKD considering gender adjusted by age

	Gender#			
Domains SF-36	Female	Male	Total	P*
	*N* = 87	*N* = 83	*N* = 170	
Functional capacity	49.08 (± 28.34)	57.10 (± 26.12)	52.99 (± 27.49)	0.018
Physical aspects	38.89 (± 37.28)	51.20 (± 38.23)	44.90 (± 38.14)	0.040
Pain	55.51 (± 27.55)	65.77 (± 28.28)	60.52 (± 28.30)	0.025
General health	54.08 (± 25.87)	54.80 (± 26.13)	54.43 (± 25.92)	0.923
Vitality	53.68 (± 24.16)	60.12 (± 21.89)	56.82 (± 23.24)	0.090
Social aspects	74.89 (± 27.68)	77.14 (± 27.93)	75.99 (± 27.75)	0.935
Emotional aspects	50.58 (± 43.12)	54.71 (± 38.01)	52.60 (± 40.64)	0.552
Mental health	60.25 (± 23.61)	69.54 (± 22.15)	64.79 (± 23.31)	0.017

#*P* = 0.016 (Wilks’ Lambda)

*Mancova

As summarized in Table 3, there was no difference in QOL among the 5 stages of CKD in pre-dialysis.

**Table 3:** Different domains of SF-36 obtained from 170 patients with CKD considering CKD staging adjusted by gender and age

		Stages					
Domains SF-36	TFG > 90	Mild	Moderate	Severe	Terminal	Total	P*
	*N* = 3	*N* = 15	*N* = 56	*N* = 64	*N* = 32	*N* = 170	
Functional capacity	75.00 (±26.45)	45.33 (±24.74)	52.32 (±27.91)	55.22 (±28.12)	51.25 (±26.85)	52.99 (±27.49)	0.546
Physical aspects	83.33 (±28.86)	38.33 (±41.04)	49.11 (±37.52)	41.53 (±37.01)	43.75 (±40.16)	44.9 (±38.14)	0.300
Pain	66.33 (± 30.60)	54.53 (± 22.53)	66.07 (± 30.71)	58.12 (± 29.39)	57.84 (± 23.49)	60.52 (± 28.30)	0.542
General health	63.00 (±37.32)	45.27 (±22.16)	56.39 (±26.62)	55.16 (±27.50)	53.03 (±22.37)	54.43 (±25.92)	0.637
Vitality	65.00 (±25.98)	51.00 (±23.69)	57.77 (±23.89)	56.25 (±23.68)	58.28 (±21.61)	56.82 (±23.24)	0.762
Social aspects	100.00 (±0.00)	71.63 (±27.74)	79.00 (±29.67)	75.28 (±27.10)	71.94 (±26.38)	75.99 (±27.75)	0.402
Emotional aspects	88.90 (±19.22)	47.35 (±47.85)	58.33 (±38.84)	49.47 (±40.33)	47.88 (±41.47)	52.60 (±40.64)	0.357
Mental health	69.33 (±32.5)	62.53 (±25.79)	65.80 (±25.00)	63.91 (±23.04)	65.41 (±19.95)	64.79 (±23.31)	0.934

*P = 0.961 (Wilks’ Lambda)

Mancova

Table 4 shows that the influence of hemoglobin on QOL was observed only in the social aspects domain, which was significantly lower in CKD patients who had hemoglobin less than or equal to 9 g/dl.

**Table 4:** Different domains of SF-36 obtained from 170 patients with CKD considering hemoglobin (Hb) adjusted by gender and age

	Level of hemoglobin*				
SF-36 domains	Hb ≤ 9.0	Hb 9.1–12.9	Hb ≥ 13	Total	P#
	*N* = 12	*N* = 56	*N* = 93	*N* = 161	
Functional capacity	46.67 (± 31.50)	48.51 (± 26.90)	56.28 (± 27.13)	52.99 (± 27.49)	0.166
Physical aspects	33.33 (± 34.23)	35.96 (± 35.98)	51.32 (± 38.74)	44.90 (± 38.14)	0.072
Pain	46.25 (± 27.30)	59.56 (± 26.51)	62.75 (± 29.11)	60.52 (±28.30)	0.270
General health	46.42 (± 19.27)	57.96 (± 24.93)	53.39 (± 27.03)	54.43 (± 25.92)	0.315
Vitality	52.92 (± 20.83)	55.00 (± 24.23)	58.32 (± 23.02)	56.82 (± 23.24)	0.819
Social aspects	51.17 (± 29.39)	78.11 (± 23.48)	77.74 (± 28.59)	75.99 (± 27.75)	0.015
Emotional aspects	47.17 (± 38.89)	43.85 (± 40.94)	58.18 (± 40.09)	52.60 (± 40.64)	0.118
Mental health	54.67 (± 22.52)	66.39 (± 22.83)	66.78 (± 23.52)	64.79 (± 23.31)	0.424

*P = 0.057 (Wilks’ Lambda)

#Mancova

Age influenced the QOL of CKD patients in the functional capacity and social aspects domains. Functional capacity was better in CKD patients aged less than 47 years. The social aspect was better in the elderly compared to young people aged up to 47 years (Table 5).

**Table 5:** Different domains of SF-36 obtained from 170 patients with CKD considering age adjusted by gender

	Age (years)#				
SF-36 domains	≤47	48–59	≥60	Total	P*
	*N* = 43	*N* = 44	*N* = 83	*N* = 170	
Functional capacity	61.28 (± 25.49)	46.36 (± 26.70)	52.22 (± 28.12)	52.99 (± 27.49)	0.015
Physical aspects	41.28 (± 35.72)	46.02 (± 41.74)	46.18 (± 37.70)	44.90 (± 38.14)	0.937
Pain	61.33 (± 25.05)	56.02 (± 26.14)	62.48 (± 30.91)	60.52 (± 28.30)	0.466
General health	54.23 (± 24.64)	48.98 (± 24.63)	57.42 (± 27.03)	54.43 (± 25.92)	0.224
Vitality	53.60 (± 21.63)	56.82 (± 22.90)	58.49 (± 24.29)	56.82 (± 23.24)	0.746
Social aspects	65.79 (± 26.75)	78.14 (± 26.07)	80.14 (± 28.09)	75.99 (± 27.75)	0.021
Emotional aspects	46.51 (± 39.99)	57.81 (± 42.49)	52.99 (± 40.05)	52.60 (± 40.64)	0.472
Mental health	60.49 (± 22.22)	67.20 (± 21.89)	65.73 (± 24.54)	64.79 (± 23.31)	0.555

#*P* = 0.011 (Wilks’ Lambda)

*Mancova

As detailed in Table 6, income had a major influence on QOL; 6 out of 8 domains investigated by the SF36 were influenced by it. In this sample, CKD patients with an income greater than 5.1 times the minimum wage had better QOL in terms of physical functioning, pain and social aspects than those who earned only one minimum wage. Physical and emotional role and mental health were also better in those whose income was greater than 5.1 times the minimum wage.

**Table 6:** Different domains of SF-36 obtained from 170 patients with CKD considering income adjusted by gender and age

	Family income*				
SF-36 domains	Up to 1 minumum wage	1.1 to 5 minimum wages	More than 5.1 minimum wages	Total	P#
	*N* = 50	*N* = 77	*N* = 42	*N* = 169	
Functional capacity	46.20 (± 27.63)	52.99 (± 26.63)	62.09 (± 27.30)	52.86 (± 27.52)	0.042
Physical aspects	32.00 (± 37.12)	42.64 (± 34.38)	63.89 (± 38.27)	44.57 (± 38.01)	0.001
Pain	54.46 (± 27.37)	59.13 (± 28.63)	71.38 (± 27.03)	60.28 (± 28.22)	0.029
General health	52.42 (± 24.08)	51.92 (± 26.89)	60.69 (± 25.26)	54.33 (± 25.96)	0.153
Vitality	53.10 (± 22.60)	56.82 (± 23.42)	61.22 (± 22.89)	56.75 (± 23.28)	0.465
Social aspects	66.99 (± 31.50)	77.49 (± 27.38)	84.23 (± 20.20)	75.85 (± 27.77)	0.013
Emotional aspects	39.97 (± 42.08)	49.77 (± 38.53)	71.35 (± 35.57)	52.32 (± 40.59)	0.001
Mental health	58.20 (± 23.91)	63.53 (± 22.66)	74.49 (± 20.41)	64.65 (± 23.31)	0.012

*P = 0.017 (Wilks’ Lambda)

#Mancova

Level of education did not influence QOL (Table 7). However, in isolation, CKD patients who had over 11 years of study had better physical functioning than those who had studied for three years at most.

**Table 7:** Different domains of SF-36 obtained from 170 CKD patients considering level of education and years of study adjusted by gender and age

	Years of study#				
Domains SF-36	Up to 3	From 3,1 to 11	More than 11	Total	P*
	*N* = 49	*N* = 62	*N* = 57	*N* = 168	
Functional capacity	45.92 (± 26.19)	51.94 (± 28.39)	60.26 (± 26.22)	52.64 (± 27.45)	0.128
Physical aspects	37.24 (± 38.56)	43.48 (± 35.37)	53.07 (± 39.82)	44.90 (± 38.14)	0.070
Pain	60.59 (± 28.37)	57.36 (± 28.35)	64.00 (± 28.26)	60.52 (± 28.30)	0.390
General health	55.12 (± 27.21)	52.77 (± 27.15)	55.70 (± 23.64)	54.43 (± 25.92)	0.604
Vitality	57.86 (± 24.10)	56.02 (± 23.65)	56.84 (± 22.37)	56.82 (± 23.24)	0.936
Social aspects	77.57 (± 29.06)	76.16 (± 28.49)	74.45 (± 26.10)	75.99 (± 27.75)	0.894
Emotional aspects	44.88 (± 41.74)	57.83 (± 39.99)	53.36 (± 40.11)	52.60 (± 40.64)	0.219
Mental health	62.78 (± 24.05)	62.75 (± 25.06)	68.81 (± 20.31)	64.79 (± 23.31)	0.147

#*P* = 0.244 (Wilks’ Lambda)

*Mancova

## Discussion

Chronic kidney disease is a serious public health issue, and the affected patients have a poor quality of life from the early stages to the evolution to dialysis or kidney transplantation. Quality of life measurement is increasingly recognized as an important tool to assess progress and determine which intervention measures can lead to better well-being and success in treatment. Several studies have shown that quality of life is impaired in chronic kidney disease at its various stages of treatment. In addition, psychological factors, anxiety, depression and alexithymia may be related to treatment complications and lower quality of life in chronic renal failure patients [15–17]. Patients undergoing kidney transplant appear to have better quality of life than those who are on dialysis, despite suffering the effect of the use of immunosuppressive drugs and being subject to infectious complications and tumors [18]. Conversely, patients in the pre-dialysis stage, despite not being dependent on the machine or dialysis, suffer from various comorbidities such as anemia, mood swings and social conditions that may interfere with treatment and their well-being, which, in turn, affect their quality of life. The results of this study indicate that, among the factors investigated, sex (*p* = 0.016), age (*p* = 0.011) and family income (*p* = 0.017) influenced the quality of life of patients with CKD. Also, CKD severity did not impact the various domains of SF-36 (*p* = 0 961) in unexpected ways.

As expected, symptoms resulting from complications and comorbidities are more common as kidney disease progresses, resulting in lower scores of quality of life. One explanation for the results observed in this study regarding the little influence of disease staging on quality of life is the small number of patients in each stage of the disease. The difference in QOL among the stages of CKD was more frequently identified in studies of a large number of individuals, generally over 1000 [10]. Nonetheless, socio-demographic variables were predictors of QOL even in cross-sectional studies that involved fewer than 1000 subjects.

In this study, the physical dimension was the most affected in CKD, as compared to the mental domains. These results are consistent with those of previous studies that also showed that the physical aspects of CKD patients are more affected in terms of QOL [8, 19].

Physical functioning was better in CKD patients aged less than 47 years, and the social aspect was better in the elderly as compared to young individuals aged up to 47 years. A younger age has been associated with a greater decline in mental health and emotional role, but with better physical functioning and general health [20]. Another research study found worse mental health in the youngest age group and worse physical functioning in the oldest age group [21]. Therefore, our results are similar to findings from other studies which identified worse physical performance but better mental health in older people [22]. A suggestion for these two groups would be to raise their awareness about the disease, making it more tolerable both physically and emotionally for patients and their families [16].

Men with CKD in this sample had higher income and higher hemoglobin, and this could explain their better QOL in terms of physical functioning, mental health, and pain in relation to women. This was in agreement with another study which showed that men and individuals with a higher income had better mental health [22].

Men obtained significantly higher scores in all scales (except physical role), including PS (Physical Summary) and MS (Mental Health Summary), while women had the worst QOL [19]. These findings may be explained by the fact that women are more prone to suffering from depression [15, 16], they experience greater pain in chronic diseases [20], and take on greater responsibility to adapt their lives to their new CKD reality [23].

Men with CKD had a higher 10-year cardiovascular risk as compared to women, probably because they were older.

In this study, the level of hemoglobin (Hb) showed no significant differences in terms of QOL, despite having negatively influenced the social aspect when below 9 g/dl. Other findings indicated that the severity of anemia had a modest effect on QOL [20, 24-26]. One possible explanation for this discrepancy may be that the majority of our sample had more than one year of outpatient follow-up and Hb > 11 g/dL [27, 28].

Level of education did not significantly influence QOL. However, CKD patients with more than 11 years of education had better physical functioning than those who had studied for three years at most. The lack of statistical significance for the relationship between level of education and QOL could be explained by the results of similar studies which found a greater PS in individuals with a higher level of education, but who were professionally active [22]. This did not occur in our research study.

CKD patients with an income greater than 5.1 times the minimum wage had better QOL in terms of physical functioning, pain, social aspects, mental health, and physical and emotional aspects, that is, they had better QOL than those who had a monthly income inferior to 5.1 wages. Unemployment and low income [16] were significantly associated with depression, which could explain the worse QOL found in our work. Depression symptoms and worse socioeconomic conditions in CKD patients have been associated with a worse QOL [29-31]. Research on end-stage renal disease patients has shown that the severity of depression symptoms is also influenced by monthly income [15].

Few studies have evaluated the influence of income on QOL. Unemployment, a low level of education and low income have been associated with worse QOL [19]. Employment, level of education, household income, physical activity, and male gender were, independently, significant predictors of PS. Employment, age and male gender were predictors of MS [19]. In a cross-sectional study, unemployment was associated with a worse QOL, and better social support and coping skills with a better QOL [31]. Another study found better mental health in people with higher income [21]. Social support is a way to offer better health care to end-stage renal disease patients [16]. In any case, a higher income may allow more leisure time or better social support. In addition, vitality in CKD patients on conservative treatment has already been associated with the “going-out frequency” [32]. These results are consistent with the evidence that socio-demographic factors are important predictors of QOL in CKD patients.

In addition, renal disease is associated with dietary, water and social restrictions, which make treatment acceptance difficult and may cause loss of QOL. It has been observed that psychosocial factors such as finance (69.3 %), logistics (66.0 %) and lack of social support (17.0 %) negatively influence the acceptance of treatment by CKD patients [33]. Moreover, CKD limits the ability to work and increases the costs related to the disease, and this also explains the worst QOL in patients with a lower income. In CKD patients already in dialysis phase, the physical component of QOL was greater in the higher income group [34].

In this study, detailed information about the sources of income showed that only a minority (28 %) still had a job (professionally active) and 50.58 % had as a source of income (which was not always exclusive) retirement or disability insurance. In chronic renal patients undergoing hemodialysis, we found low scores for aspects of QOL associated with “job status”, “cognitive function”, and “physical and emotional roles”, while high scores were obtained in terms of “patient satisfaction”, “support from the dialysis staff”, and “quality of social interaction” [35].

Greater attention to the level of habitual activity among patients with CKD and ESRD could help these individuals to keep their jobs, which could improve their overall QOL [36]. Other studies have also strengthened the relationship between a number of demographic characteristics in QOL [19, 33], so much so that the need to offer CKD patients on conservative treatment more opportunities for self-management has been investigated, addressing issues related to social disadvantage [37].

The economic conditions of low-income families affected by renal disease reinforce the interrelations between chronic disease, economic well-being and quality of life in this population. However, since CKD is highly interactive with multiple comorbidities, health costs for these patients cannot be attributed solely to CKD. Indeed, a financial policy for the pre-dialysis period can be a suggestion for better results in terms of QOL [7].

Health assistance is a social right for all citizens and it requires the direct and positive intervention of the State in order to provide services for the diagnosis, treatment and medical follow-up of diseases, including the improvement of patients’ QOL. Low-income patients depend on public transportation to go to health units; however, public transportation is not always reliable, and this causes them stress and fatigue, jeopardizing the outpatient follow-up of diseases. In addition, low-income patients are more worried about the impact of the disease on production for survival, leading to family conflicts and the consequent development of comorbidities, such as depression, which impact QOL. Leisure activities are jeopardized for low-income patients. Previous studies have already shown that functional independence, high-income families and absence of depression are related to better QOL [29, 30, 36]. In view of the fact that equity is one of the universal principles of the national health system in Brazil, it is suggested that relevant programs be offered to the low-income population with chronic renal disease.

## Conclusions

In this study, a worse QOL was observed in CKD patients since the early stages of the disease, but a significant relationship between CKD staging and the SF-36 domains was not identified. Gender and age influenced QOL, but family income was the aspect that had the most influence. These findings suggest that many efforts should be made to reduce the effect of these economic factors on the QOL of CKD patients. They also reinforce the need for longitudinal and intervention studies of pre-dyalitical CKD patients.

### Limitations

The selected sample included only patients who were already being monitored in specialized outpatient clinics, and this limits the conclusions drawn based on the findings to the population studied. The possible influence of time and treatment on the evolution of QOL was not evaluated longitudinally in this cross-analytical study.
